# Contributions of HA1 and HA2 Subunits of Highly Pathogenic Avian Influenza Virus in Induction of Neutralizing Antibodies and Protection in Chickens

**DOI:** 10.3389/fmicb.2020.01085

**Published:** 2020-06-05

**Authors:** Edris Shirvani, Anandan Paldurai, Berin P. Varghese, Siba K. Samal

**Affiliations:** Virginia-Maryland College of Veterinary Medicine, University of Maryland, College Park, College Park, MD, United States

**Keywords:** highly pathogenic avian influenza virus, hemagglutinin subunits, protective epitopes, native conformation, poultry vaccines, natural host model

## Abstract

Highly pathogenic avian influenza virus (HPAIV) subtype H5N1 causes a devastating disease in poultry. Vaccination is an effective method of controlling avian influenza virus (AIV) infection in poultry. The hemagglutinin (HA) protein is the major determinant recognized by the immune system of the host. Cleavage of the HA precursor HA0 into HA1 and HA2 subunits is required for infectivity of the AIV. We evaluated the individual contributions of HA1 and HA2 subunits to the induction of HPAIV serum neutralizing antibodies and protective immunity in chickens. Using reverse genetics, recombinant Newcastle disease viruses (rNDVs) were generated, each expressing HA1, HA2, or HA protein of H5N1 HPAIV. Chickens were immunized with rNDVs expressing HA1, HA2, or HA. Immunization with HA induced high titers of serum neutralizing antibodies and prevented death following challenge. Immunization with HA1 or HA2 alone neither induced serum neutralizing antibodies nor prevented death following challenge. Our results suggest that interaction of HA1 and HA2 subunits is necessary for the display of epitopes on HA protein involved in the induction of neutralizing antibodies and protection. These epitopes are lost when the two subunits are separated. Therefore, vaccination with either a HA1 or HA2 subunit may not provide protection against HPAIV.

## Introduction

Avian influenza (AI) is a serious disease in poultry. Avian influenza virus (AIV) belongs to the genus *Alphainfluenzavirus* in the family *Orthomyxoviridae* (International Committee on Taxonomy of Viruses [ICTV], 2018). Avian influenza viruses are divided into subtypes based on antigenic differences in the two major surface glycoproteins, hemagglutinin (HA) and neuraminidase (NA) (Palese and Shaw, 2013). Currently, 16 HA subtypes (H1–H16) and 11 NA subtypes (N1–N11) in different combinations have been found in avian populations. Avian influenza viruses are classified into highly pathogenic avian influenza (HPAI) and low pathogenic avian influenza (LPAI) viruses. Highly pathogenic avian influenza viruses in poultry are of H5 and H7 subtypes (Palese and Shaw, 2013; OIE, 2018b). Furthermore, in recent years, H5, H7, H9, and H10 subtypes of AIVs have caused human infections, with H5 and H7 causing serious disease in humans (Chen et al., 2014; Sutton, 2018; Peacock et al., 2019).

Vaccination is considered a practical method to control AI in endemic countries (Swayne, 2012; OIE, 2018b). However, live vaccines against AIV are not recommended for use in poultry, because of the potential risk of genome segment reassortment between vaccine and field strains that can lead to the emergence of viral variants with different antigenic properties (OIE, 2018b). The vast majority of AIV vaccines (95.5%) currently used in the field are inactivated vaccines (Swayne, 2012). However, they do not elicit strong immune responses. The process of production and administration of this type of vaccine are expensive, labor intensive, and time consuming. Furthermore, vaccination programs with inactivated vaccines cause difficulty as it is hard to differentiate vaccinated birds from infected ones, which can result in trade restrictions (Swayne, 2006; Swayne et al., 2011). Therefore, viral vectored vaccines containing the HA protein of highly pathogenic avian influenza virus (HPAIV) have been developed and licensed in some countries (OIE, 2018b; Suarez and Pantin-Jackwood, 2017).

Hemagglutinin is an important multifunctional protein in influenza virus. The HA protein mediates binding of the virus to the host cell receptor. It is the major antigen against which neutralizing antibodies are induced during infection (Palese and Shaw, 2013). Each HA monomer is synthesized as an inactive precursor protein HA0. The cleavage of HA0 protein into HA1 and HA2 subunits by a cellular protease is a prerequisite for viral infectivity (Klenk et al., 1975; Lazarowitz and Choppin, 1975). Following cleavage, the HA1 and HA2 subunits are held together by disulfide bonds. The HA1 binds to sialic acid receptors on cell surfaces and initiates virus entry by endocytosis. Due to acidic pH-induced conformational change of HA2 in endosomes, the fusion of viral and endosomal membranes occurs (Palese and Shaw, 2013). The HA1 subunit, which forms the globular head, is highly variable among subtypes and contains major neutralizing epitopes. The HA2 subunit, which forms most of the stalk domain, is considerably conserved among subtypes and contains few but cross-reactive neutralizing epitopes (Palese and Shaw, 2013; Kirkpatrick et al., 2018; Koutsakos et al., 2019).

The presence of subtype-specific neutralizing epitopes on a HA1 subunit and the presence of broad neutralizing epitopes on a HA2 subunit makes them potential candidates to develop subtype-specific and universal vaccines against influenza viruses, respectively (Palese and Shaw, 2013; Koutsakos et al., 2019). To date, several head-based and stalk-based vaccine candidates against human influenza viruses have been evaluated in mice and/or ferret models, but an effective vaccine is not yet available (Gao et al., 2006; Steel et al., 2010; Krammer et al., 2014; Mallajosyula et al., 2014; Yassine et al., 2015; Sutton et al., 2017; Krammer and Palese, 2019). Some promising outcomes from mice and/or ferret model challenge studies keep these approaches attractive (Gao et al., 2006; Steel et al., 2010; Mallajosyula et al., 2014; Yassine et al., 2015). Although mice and ferrets represent invaluable animal models to study human influenza virus pathogenesis, they may not accurately reflect the disease pathogenesis in humans (Thangavel and Bouvier, 2014; Zeng et al., 2019); hence, mice and ferrets may not be suitable animal models to evaluate the protective efficacies of HA1 and HA2 subunit vaccines against an influenza virus challenge. Therefore, there is a need to clearly evaluate the contributions of HA1 and HA2 subunits in a natural host model. Chickens are highly susceptible to H5N1 HPAIV infection and play a critical role in the spread of the virus. Hence, chickens provide a natural model to determine the contributions of HA1 and HA2 subunits of H5N1 HPAIV in the induction of neutralizing antibodies and protection. Viral vectored vaccines containing the intact HA protein of H5N1 HPAIV have been investigated in chickens in several studies (Kim and Samal, 2016, 2019; Suarez and Pantin-Jackwood, 2017). However, the individual contributions of HA1 and HA2 subunits of HPAIV HA protein in the induction of neutralizing antibodies and protection has not been investigated in chickens.

Newcastle disease virus (NDV) (Veits et al., 2006; Kim and Samal, 2016, 2019), turkey herpesvirus (HVT) (Balzli et al., 2018), fowl pox virus (FPV) (Bublot et al., 2007), adenovirus (Toro and Tang, 2009; Steel et al., 2010), infectious laryngotracheitis virus (ILTV) (Pavlova et al., 2009), and Marek’s disease virus (MDV) (Cui et al., 2013) have been used as live recombinant vaccine vectors against HPAIV in chickens. Among these vectors, NDV is most promising, because it infects chickens through the respiratory tract as HPAIV, resulting in eliciting robust local and systemic immune responses (Perez et al., 2019). Newcastle disease virus belongs to the genus *Orthoavulavirus* in the family *Paramyxoviridae* (International Committee on Taxonomy of Viruses [ICTV], 2018). A virulent NDV strain, LaSota, has been used as a safe and effective live vaccine for more than 60 years (Samal, 2019). In this study, we have used reverse genetics techniques to generate recombinant NDVs (rNDVs) expressing HA1, HA2, or HA protein of H5N1 HPAIV. The individual contributions of HA1 and HA2 subunits of HPAIV HA protein in the induction of neutralizing antibodies and protection were evaluated in chickens. Our results showed that when the HA1 and HA2 subunits were separated neither provided protection nor induced neutralizing antibodies. Therefore, rNDVs expressing HA1 or HA2 subunits were not protective in chickens against HPAIV challenge, while rNDV expressing the intact HA protein provided complete protection. These results may have implications in the development of vaccines against all influenza viruses.

## Materials and Methods

### Cells and Viruses

Human epidermoid carcinoma (HEp-2) cells, chicken embryo fibroblast (DF1) cells, and Madin-Darby canine kidney (MDCK) cells purchased from the American Type Culture Collection (ATCC, Manassas, VA), were used to recover rNDVs, to determine *in vitro* characteristics of rNDVs, and to assess neutralizing antibodies induced against AIV, respectively. HEp-2 and DF1 cells were grown in Dulbecco’s minimal essential medium (DMEM) containing 10% fetal bovine serum (FBS). Madin-Darby canine kidney cells were grown in DMEM containing 5% FBS. The rNDV strain LaSota and rNDVs expressing HA1, HA2, or HA protein of H5N1 HPAIV, recovered by reverse genetic technique, were grown in 10-day-old embryonated specific pathogen free (SPF) chicken eggs (Charles Rivers, MA) at 99.5°F. The HPAIV strain A/Vietnam/1203/2004 (H5N1) and the LPAIV strain A/Mallard/Pennsylvania/10218/1984 (H5N2) viruses were propagated in 10-day-old SPF embryonated chicken eggs in Biosafety level-3 plus and Biosafety level-2 facilities, respectively. The 50% endpoint titers of H5N1 HPAIV and H5N2 LPAIV in allantoic fluid harvested from eggs were determined by 50% embryo infectious dose (EID_50_) and tissue culture 50% infectious dose (TCID_50_) methods, respectively. The titers of viruses were calculated using a formula derived from Reed and Muench and Spearman–Karber methods (Ramakrishnan, 2016). The modified vaccinia virus strain Ankara, expressing T7 RNA polymerase (MVA-T7), was propagated using primary chicken embryo fibroblast cells.

### Generation of rNDVs Expressing HA1, HA2, or HA Protein of HPAIV (H5N1)

**FIGURE 1:**
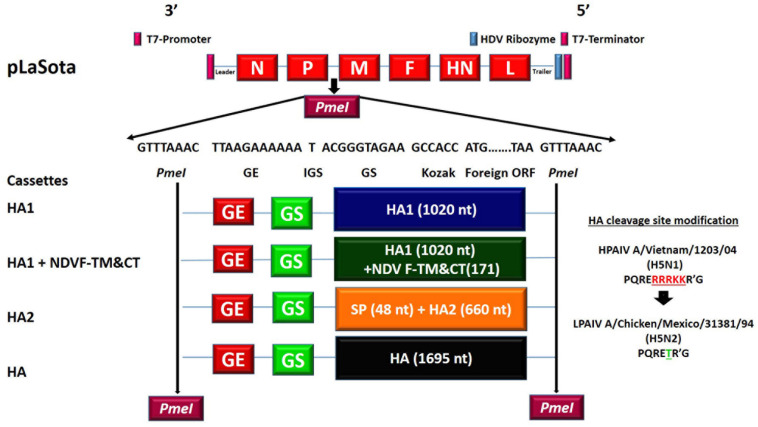
Construction of recombinant NDVs containing H5N1 HPAIV HA1, HA2, or HA gene. The following four transcription cassettes were inserted into individual plasmids containing cDNA of LaSota between P and M genes using *Pme*I site: (i) codon optimized HA1 subunit of HA gene of H5N1 HPAIV (1,020 nt), (ii) codon optimized HA1 subunit of HA gene of H5N1 HPAIV (1,020 nt) fused with N-terminus of transmembrane and cytoplasmic tail of F gene of NDV (171 nt), (iii) the N-terminus of codon optimized HA2 subunit of HA gene of H5N1 HPAIV (660 nt) fused with C-terminus of signal peptide sequence of HA gene of H5N1 HPAIV(48 nt), and (iv) the codon optimized HA gene of H5N1 HPAIV (1;1695 nt). Each transcription cassette contains; *Pme*I restriction enzyme site sequence at two ends of the transcription cassette plus in the constant order; 3′-GE signal of NDV, one T nucleotide as intergenic sequence (IGS), GS signal of NDV, Kozak sequence (In order to follow the rule of six ACC and GCCACC were used for HA construct and other constructs, respectively.), and foreign gene ORF sequence-5′.

A plasmid pBR322 containing full-length antigenomic cDNA of NDV strain LaSota had been constructed previously (Huang et al., 2001). A previously constructed plasmid of full-length antigenomic cDNA of NDV strain LaSota containing the transcription cassette of HPAIV A/Vietnam/1203/2004 (H5N1) HA gene between NDV P and M genes also was used in this study (Kim et al., 2014). The polybasic cleavage site (PQRERRRKKR’G) of HPAIV strain A/Vietnam/1203/2004 (H5N1) was modified to the monobasic cleavage site (PQRETR’G) of LPAIV strain A/Mexico/31381/94 (H5N2). The HA1, HA2, and HA genes were codon optimized for higher levels of expression. Four transcription cassettes were designed: (i) a transcription cassette containing the HA1 subunit (1020 nt) to determine the contribution of the HA1 subunit in the induction of neutralizing antibodies and protection, (ii) a transcription cassette containing the HA1 subunit (1020 nt) fused with TM and CT of NDV F gene (171 nt) for incorporation into NDV envelope, (iii) a transcription cassette containing the signal sequence of the HA gene (48 nt) fused with the HA2 gene (660 nt) to determine the contribution of the HA2 subunit in the induction of neutralizing antibodies and protection, and (iv) a transcription cassette containing the HA gene, constructed previously (Kim et al., 2014), to evaluate the contributions of HA1 and HA2 subunits in comparison with intact HA in the induction of protection and immunity. The transcription cassettes of codon optimized HA1 and HA2 genes were amplified from the plasmid containing the codon optimized HA gene of HPAIV A/Vietnam/1203/2004 (H5N1). The amplified fragments were cloned into individual pGEM^®^-T Easy Vectors (Promega Corporation). The correct sequence of the genes introduced into the pGEM^®^-T vectors was confirmed by nucleotide sequence analysis. Then, they were re-cloned into complete individual plasmids containing cDNA of NDV strain LaSota at P and M genes junction using *Pme*I site, following digestion of transcription cassettes from shuttle vectors using *Pme*I restriction enzyme (Figure 1). Recombinant Newcastle disease viruses containing the HA1 gene, the HA1 gene fused with TM and CT of NDV F gene, signal peptide fused with the HA2 gene, and the intact HA gene were recovered by reverse genetics as described previously (Huang et al., 2001). The presence of transcription cassettes containing HPAIV genes in rNDVs genomes was confirmed by RT-PCR. Briefly, a primer pair set (NDV strain LaSota P gene 2899 forward primer: 5′ TTAAACCCGCCACTGCATGCGG 3′ and NDV strain LaSota M gene 3297 reverse primer: 5′ CAGCCCAATTGTCCTAGATG 3′) was used to amplify the transcription cassettes containing HPAIV genes from cDNA synthesized from extracted rNDVs genome. Thirty-five cycles of PCR at 94°C for 30 s of denaturation, 56°C for 30 s of annealing, and 68°C for 150 s of elongation using the TAKARA LA Tag polymerase was used to amplify the fragments.

### Expression of HA1, HA2, and HA Proteins of H5N1 HPAIV

The expression of HA1, HA2, and HA proteins were detected in DF1 cells by Western blot analysis. DF1 cells were infected with rLaSota, rLaSota/HA1, rLaSota/HA1 + NDV F TM&CT, rLaSota/HA2, and rLaSota/HA at a multiplicity of infection (MOI) of 0.1 in presence of 10% fresh allantoic fluid, harvested from embryonated SPF chicken eggs. DF1 cell lysates and supernatants were collected 30 h post-infection. A polyclonal chicken anti H5N1 HPAIV and a monoclonal anti NDV HN protein sera were used to detect the expression of HA1, HA2, and HA proteins of HPAIV and HN protein of NDV by Western blot analysis, respectively. The surface expression of HA1, HA2, and HA proteins by NDV vectors was confirmed by an indirect immunofluorescence assay (IFA). Monolayer of DF1 cells on four-well chamber slides were infected with rNDVs. The cells were fixed and blocked on the slide surface 24 h post infection using 3% paraformaldehyde and 3% goat serum, respectively. The expression of HPAIV on the cell surface was detected using the above polyclonal chicken anti H5N1 HPAIV primary antibody and a goat anti chicken fluorescent conjugated secondary antibody (KPL). The expression of HA1, HA2, and HA proteins in embryonated chicken eggs and the incorporation of HA1, HA2, and HA proteins into an NDV envelope were assessed by Western blot analysis. Ten-days-old embryonated SPF chicken eggs were infected with rLaSota, rLaSota/HA1, rLaSota/HA1 + NDV F TM&CT, rLaSota/HA2, or rLaSota/HA. Allantoic fluids were harvested from infected eggs three days after infection. Allantoic fluid also was collected from uninfected eggs as control. The allantoic fluids were centrifuged at 1500 rpm for 10 min, then were centrifuged through 30% sucrose cushion (SW41rotor) at 25,000 rpm for 2.5 h at 4°C. Then pellets were dissolved in 50 μl PBS, lysed, and analyzed by Western blot using the two above antisera.

### Growth Characteristics of rNDVs Expressing HA1, HA2, or HA Protein of H5N1 HPAIV

Recombinant Newcastle disease viruses expressing HPAIV HA1, HA2, or HA protein were passaged in eggs. The allantoic fluids containing high titers (2^8^ HAU/50 μl) of each recombinant virus were aliquoted in vials and stored at −70°C. The titer of each virus was determined by plaque forming unit (PFU) assay on DF1 cells in the presence of 10% fresh allantoic fluid. Monolayer DF1 cells in 6-well tissue culture plates were washed with DMEM and infected at an MOI of 0.1 with parental rLaSota, rLaSota strains expressing HA1, HA1 + NDV F TM&CT, HA2, or HA protein. After 90 min adsorption at 37°C, cells were washed with DMEM, then incubated with DMEM containing 10% fresh SPF chicken egg allantoic fluid at 37°C in presence of 5% CO_2_. At intervals of 8 h until 64 h post-infection, volumes of 200 μl of supernatant from infected cells were collected and replaced with equal volumes of fresh DMEM containing 10% fresh allantoic fluid. The titer of rNDVs in collected supernatants was determined by TCID_50_ method on DF1 cells. The titers were calculated using a formula derived from Reed and Muench and Spearman–Karber methods (Ramakrishnan, 2016).

### The Protective Efficacies of rNDVs Expressing HA1, HA2, or HA Protein Against H5N1 HPAIV Challenge

The protective efficacies of rNDVs expressing HA1, HA2, or HA protein of H5N1 HPAIV were evaluated in SPF chicks immunized at one day old and challenged against H5N1 HPAIV at 3 weeks after immunization. A total of 50 one-day-old SPF White Leghorn chicks, obtained from Charles Rivers, were divided into five groups of 10 each in Biosafety level-2. Chicks of groups one to five were immunized with 10^6^ PFU, 200 μl in volume, of rLaSota as empty vector control, rLaSota/HA1, rLaSota/HA1 + NDV F TM&CT, rLaSota/HA2, and rLaSota/HA, respectively, via the oculonasal route. Three weeks after immunization, blood was collected from all chickens. Then, they were moved into the Biosafety level-3 plus facility and were challenged with 10^6^ EID_50_, 200 μl in volume, of HPAIV A/Vietnam/1203/2004 (H5N1) via the oculonasal route (OIE, 2018b). The infected birds were observed daily for 10 days after challenge. The mortality rate was recorded for each group daily. We conducted all experiments involving avirulent NDV strain LaSota in our USDA approved Biosafety level-2 and Biosafety level-2 plus facilities and the experiment involving HPAIV in our USDA approved Biosafety level-3 plus facility following the guidelines and approval of the Institutional Animal Care and Use Committee (IACUC), University of Maryland.

### Serological Assays

The sera were collected from all chickens three weeks after immunization. The humoral antibodies induced against NDV and HPAIV in sera of immunized chickens were assessed. Hemagglutination inhibition (HI) assay using the protocol of OIE was used to assess the level of antibody titers mounted against a LPAIV A/Mallard (H5N2) in chickens immunized with rNDVs (OIE, 2018b). To confirm the result, we repeated the HI assay four times and we calculated the titers as the mean of four experiments. The virus neutralization assay was used to measure neutralizing antibodies induced against a LPAIV A/Mallard (H5N2) in chickens immunized by rNDVs. Briefly, serum samples of all immunized chickens were incubated at 56°C for 30 min. The titer of LPAIV (H5N2) was determined by TCID_50_ on MDCK cells in the presence of DMEM containing 1μg/ml N-tosyl-l-phenylalanine chloromethyl ketone (TPCK)-treated trypsin. 100 TCID_50_ of H5N2 LPAIV was mixed with 2-fold dilutions of antiserum and incubated for 90 min at 37°C. 100 μl of each serum and virus mixture was added into the monolayer of MDCK cells, three wells of 96-well tissue culture plate per dilution and incubated at 37°C. One hour after incubation, the supernatant of wells were removed and wells were washed with DMEM. Hundred microliter of DMEM containing 1 μg/ml TPCK-treated trypsin were added to each well. The plates were incubated at 37°C in the presence of 5% CO_2_. Three days after infection, 50 μl of supernatant of each well were transferred to HA assay plate. The presence of H5N2 LPAIV in supernatants were detected by HA assay using 1% chicken RBC. The serum titers were calculated using a formula derived from Reed and Muench and Spearman–Karber methods (Ramakrishnan, 2016). Hemagglutination inhibition assay using the protocol of OIE was used to assess the level of antibody titers mounted against NDV strain LaSota in chickens immunized with rNDVs (OIE, 2018a).

### Statistical Analysis

The data for antibody response against LPAIV was analyzed by paired *t*-test between each two groups. The data for antibody response against NDV was analyzed among groups by One-Way-ANOVA analysis (Tukey test). To avoid bias, HPAIV challenge experiment was designed as a blinded study.

## Results

### Generation rNDVs Expressing HA1, HA2, or HA Protein of H5N1 HPAIV

The expression cassettes of HPAIV HA gene subunits were constructed and inserted between P and M genes of NDV in individual plasmids containing full length antigenomic cDNA of NDV strain LaSota (Figure 1). The correct sequence of transcription cassettes containing HPAIV HA subunits and flanking regions was confirmed by sequence analysis. Infectious rNDVs containing the HA1 subunit, HA1 subunit fused with NDV-F TM and CT domains, the HA2 subunit, and the HA gene of HPAIV were recovered from all cDNAs by reverse genetics technique using HEp-2 cells. They were named rLaSota/HA1, rLaSota/HA1 + NDV F TM&CT, rLaSota/HA2, and rLaSota/HA, respectively. The presence of HA1, HA2, and HA genes in rNDVs were confirmed by RT-PCR.

### Expression of HA1, HA2, and HA Proteins of H5N1 HPAIV

**FIGURE 2:**
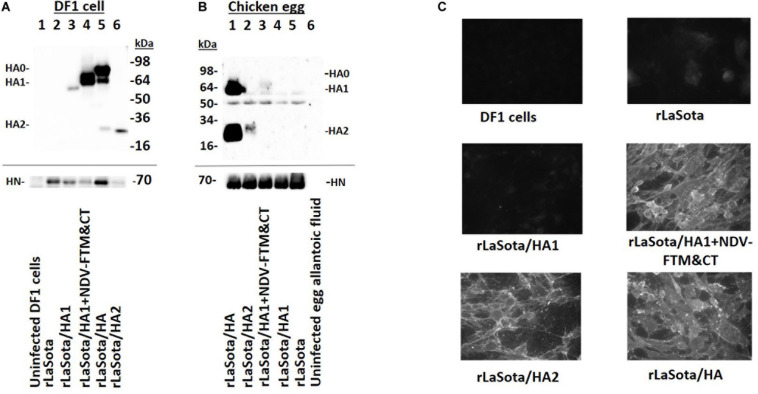
The expression of HA1, HA2, and HA proteins of HPAIV by rNDVs. **(A)** The expression of HA1, HA2, and HA proteins were detected in infected DF1 cell lysates by Western blot analysis using a chicken polyclonal anti H5N1 HPAIV serum **(upper panel)**; uninfected DF1 cells (lane 1), rLaSota (lane 2), rLaSota/HA1 (lane 3), rLaSota/HA1 + NDV F TM&CT (lane 4), rLaSota/HA (lane 5), and rLaSota/HA2 (lane 6). A monoclonal anti NDV HN antibody was used to detect the 70 kDa HN protein of NDV in DF1 cell lysates **(lower panel)**; uninfected DF1 cells (lane 1), rLaSota (lane 2), rLaSota/HA1 (lane 3), rLaSota/HA1 + NDVF TM&CT (lane 4), rLaSota/HA (lane 5) and rLaSota/HA2 (lane 6). The full length of cropped gels is presented in Supplementary Figure 1. **(B)** The expression of HA1, HA2 and HA proteins of HPAIV in embryonated chicken eggs and the incorporation of these proteins into NDV particles were detected by Western blot using a chicken polyclonal anti H5N1 HPAIV serum **(upper panel)**; rLaSota/HA (lane 1), rLaSota/HA 2(lane 2), rLaSota/HA1 + NDVF TM&CT (lane 3), rLaSota/HA1 (lane 4), rLaSota (lane 5) and uninfected allantoic fluid (lane 6). A monoclonal anti NDV HN antibody was used to detect the 70 kDa HN protein of NDV **(lower panel)**; rLaSota/HA (lane 1), rLaSota/HA 2(lane 2), rLaSota/HA1 + NDV F TM&CT (lane 3), rLaSota/HA1 (lane 4), rLaSota (lane 5) and uninfected allantoic fluid (lane 6). **(C)** DF1 cells were infected with rNDVs at MOI of 0.1. The cells were fixed with 3% paraformaldehyde 24 h post-infection, then were blocked with 3% goat serum. The florescence signals on the surface of cells were observed by immunofluorescence microscope after interacting the cells with a primary chicken polyclonal anti H5N1 HPAIV serum and a secondary FITC conjugated goat anti chicken antibody.

The expression of HA1, HA2, and HA proteins of HPAIV was detected by Western blot analysis of infected DF1 cell lysates (Figure 2A, upper panel). The HA protein was expressed efficiently (Figure 2A, lane 5), a ∼70 kDa band representing uncleaved HA protein (HA0), a ∼64 kDa band representing HA1 subunit, and a ∼25 kDa band representing HA2 subunit. The expression of HA1 subunit fused with NDV F protein TM and CT was much higher than that of HA1 subunit alone, a ∼60 kDa band represents HA1 subunit fused with NDV F protein TM and CT (Figure 2A, lane 4), and a ∼55 kDa band represents HA1 subunit (Figure 2A, lane 3). In the case of HA2 expressed by rLaSota/HA2, there is a band (∼25 kDa) on the bottom of the gel (Figure 2A, lane 6). Lanes 1 and 2 of Figure 2A represent DF1 cells and rNDV, respectively, as controls. A monoclonal anti-NDV HN antibody was used to detect a ∼70 kDa of the HN protein of NDV in cell lysates (Figure 2A, lower panel). We also evaluated expression of HPAIV HA1, HA2, and HA proteins in eggs and their incorporation into an NDV envelope (Figure 2B). Our results showed that the HA protein was expressed in eggs and incorporated into an NDV envelope, efficiently. The ∼60 and ∼25 kDa bands represent cleaved HA1 and HA2 subunits, respectively (Figure 2B, lane 1). The HA2 subunit was expressed in eggs and incorporated into the NDV envelope, inefficiently. A ∼25 kDa band shows HA2 subunit (Figure 2B, lane 2). The HA1 subunit fused with NDVF protein TM and CT showed very little expression in the egg and incorporation into NDV envelope. A very weak ∼60 kDa band represents HA1 subunit fused with NDV F protein TM and CT (Figure 2B, lane 3). Lane 4 of Figure 2B shows that HA1 without TM and CT did not express in the egg and did not get incorporated into the NDV envelope. Lanes 5 and 6 of Figure 2B represents partially purified NDV particles and allantoic fluid as control. The ∼50-55 kDa bands in lanes 1–5 were as a result of a non-specific interaction of antibodies with NDV proteins. A monoclonal anti-NDV HN antibody was used to detect the ∼70 kDa HN protein of NDV (Figure 2B, lower panel). The surface expression of HA1, HA2, and HA proteins by NDV vectors were detected by IFA. HA1 fused with NDV F TM and CT domains, HA2 and HA proteins were expressed at a high level on the surface of DF1 cells, whereas HA1 alone was not expressed on the cells’ surface (Figure 2C). In order to determine the conformational structure of expressed proteins, the expression of HA1, HA2, and HA proteins by rNDVs was detected using western blot analysis under non-reducing non-denaturing conditions. Both HA1 and HA2 in DF1 cell lysates infected with rNDVs (Supplementary Figures 2A,B) and in chicken eggs and purified NDV particles (Supplementary Figures 2C,D) were detected at sizes that were larger than our predictions. The oligomerization of HA1, HA2, and HA proteins were also determined by Western blot analysis (Supplementary Figure 3). The oligomer forms were detected for HA1, HA2, and HA proteins under non-reducing and denaturing conditions (Supplementary Figure 3A).

### Growth Characteristics of rNDVs Expressing HA1, HA2, or HA Protein of H5N1 HPAIV

**FIGURE 3:**
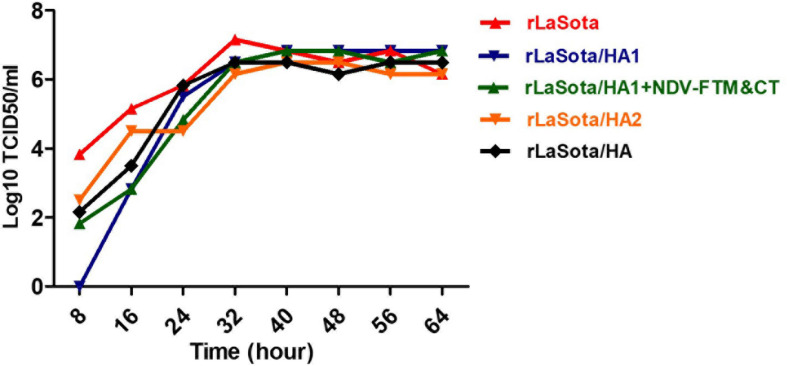
Growth kinetics of rNDVs expressing HA1, HA2, or HA protein in DF1 cells. The monolayers of DF1 cells in six-well plate tissue culture were infected with rNDVs at an MOI of 0.1. The over layered media containing the virus were removed after one-hour adsorption and replaced with fresh DMEM containing 10% allantoic fluid. The titer of viruses in volumes of supernatants collected in 8 h intervals were detected by TCID_50_ using DF1 cells.

The rNDVs expressing HA1, HA2, or HA protein were passaged in 10-day-old embryonated SPF chicken eggs. All rNDVs replicated in eggs efficiently with titers of 2^8^ HAU/_50_ μl (parental rNDV strain LaSota usually replicates in eggs with titer of 2^9^ HAU/_50_μl). The multicycle growth kinetics of rNDVs in the presence of fresh allantoic fluid, as a source of protease, in DF1 cells showed that rNDVs expressing HA1, HA2, or HA protein grew at similar levels compared to the parental rNDV. Compared to parental rNDV, other rNDVs grew slightly slower at first, but viruses reached similar titers after 32 h. However, there was no statistical significance among groups (Figure 3).

### The Protective Efficacies of rNDVs Expressing HA1, HA2, or HA Protein in Chickens Against H5N1 HPAIV Challenge

**FIGURE 4:**
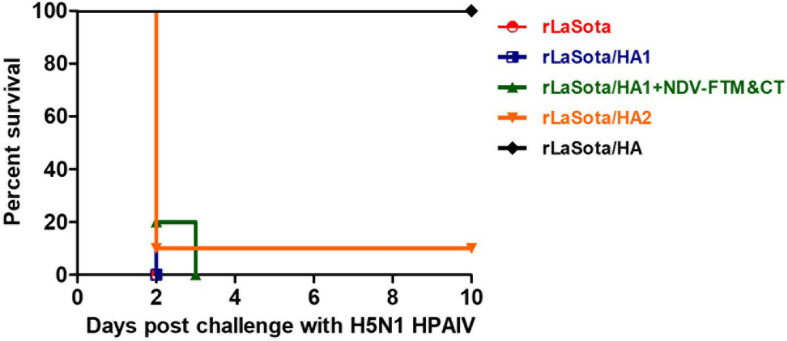
Survival rate of immunized chickens with rNDVs expressing HA1, HA2, or HA protein of HPAIV against H5N1 HPAIV challenge. Groups of ten SPF chicks were immunized with 10^6^ PFU of rLaSota, rLaSota/HA1, rLaSota/HA1 + NDV F TM&CT, rLaSota/HA2, or rLaSota/HA via the oculonasal route at one day old. Three weeks after immunization, chickens were challenged with 10^6^ EID_50_ of HPAIV A/Vietnam/1203/2004 (H5N1) through oculanasal route. Chickens were observed daily for ten days after challenge. The percent of survival rate was recorded for each group.

The protective efficacies of rNDVs expressing HA1, HA2, or HA protein were evaluated in one-day-old SPF chicks immunized at one day old and challenged with H5N1 HPAIV at three weeks post immunization. The survival rates against HPAIV were recorded daily for ten days after challenge (Figure 4). The results showed an 100% survival rate for chickens immunized with rLaSota/HA until ten days post-challenge, while an 100% mortality was observed for chickens immunized with empty LaSota vector and chicks immunized with rLaSota/HA1 at day two post-challenge. The mortality rate for chickens immunized with rLaSota/HA2 was nine out of ten chickens at day two post-challenge. One out of ten chickens immunized with rLaSota/HA2 survived until ten days post-challenge. In the case of chickens immunized with rLaSota/HA1 + NDV F TM&CT, results showed eight out of ten chickens died at day two post-challenge and the remaining two chickens died at day three post-challenge. In this study, virus shedding from infected birds was not evaluated, because all chickens immunized with empty LaSota vector, rLaSota/HA1, or rLaSota/HA2 died before day four post-challenge. However, in another challenge, we evaluated the protective efficacy of rLaSota/HA in preventing post-challenge oropharyngeal viral load in SPF chickens (Shirvani et al., 2020).

### Antibody Response Against AIV

**FIGURE 5:**
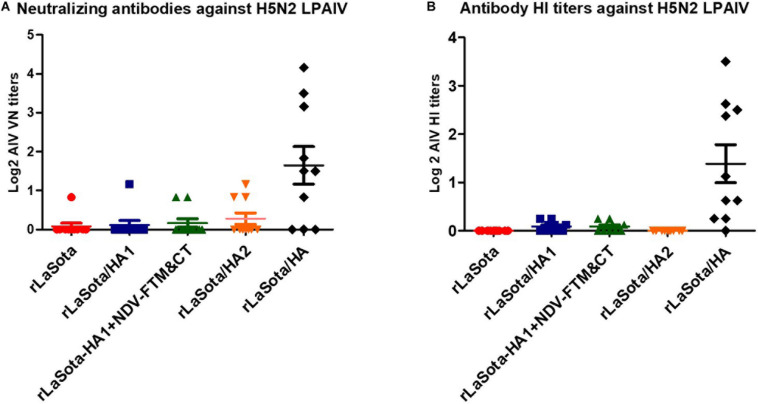
Antibody response against AIV. SPF chicks were immunized at one day old and sera were collected at day 21 after immunization. Neutralizing antibodies against a heterologous H5N2 LPAIV in collected sera were assessed by a micro-virus neutralization assay on MDCK cells using a standard protocol **(A)**. Symbols and horizontal lines represent individual titers of each sample and mean titers of each group, respectively. HI antibody titers were assessed against a heterologous H5N2 LPAIV by HI assay (repeated four times) using the protocol of OIE **(B)**. Each symbol represents mean titers of four HI assays for individual samples and horizontal lines represent mean titers of each group, respectively. Serum titers were expressed as reciprocals Log2 dilution.

Neutralizing antibodies induced against LPAIV (H5N2) in serum samples of chickens at 21 days after immunization were assessed by a micro-virus neutralization assay on MDCK cells. The results showed that compared to rLaSota group, the significant level of neutralizing antibodies against LPAIV (H5N2) were detected only in serum samples of chickens immunized with rLaSota expressing intact HA protein (*P* < 0.05). However, compared to the rLaSota group, insignificant titers of neutralizing antibodies against LPAIV (H5N2) were detected in serum samples of chickens immunized with rLaSota/HA2 and rLaSota/HA1 + NDV F TM&CT (*P* > 0.05) (Figure 5A). The data were statistically analyzed by the paired *t*-test between the rLaSota group and each vaccinated group.

The HI titers of antibodies induced against a heterologous H5N2 LPAIV were assessed in sera of chickens 21 days after immunization by HI assay using the protocol of OIE (repeated four times). The results showed that, compared to groups immunized with rLaSota, rLaSota/HA1, rLaSota/HA1 + NDV F TM&CT, or rLaSota/HA2, HI titers against H5N2 LPAIV was detected at a significantly higher level only in serum samples of chickens immunized with rLaSota expressing intact HA protein (*P* < 0.05) (Figure 5B). The data were statistically analyzed by the paired *t*-test between each two groups.

**FIGURE 6:**
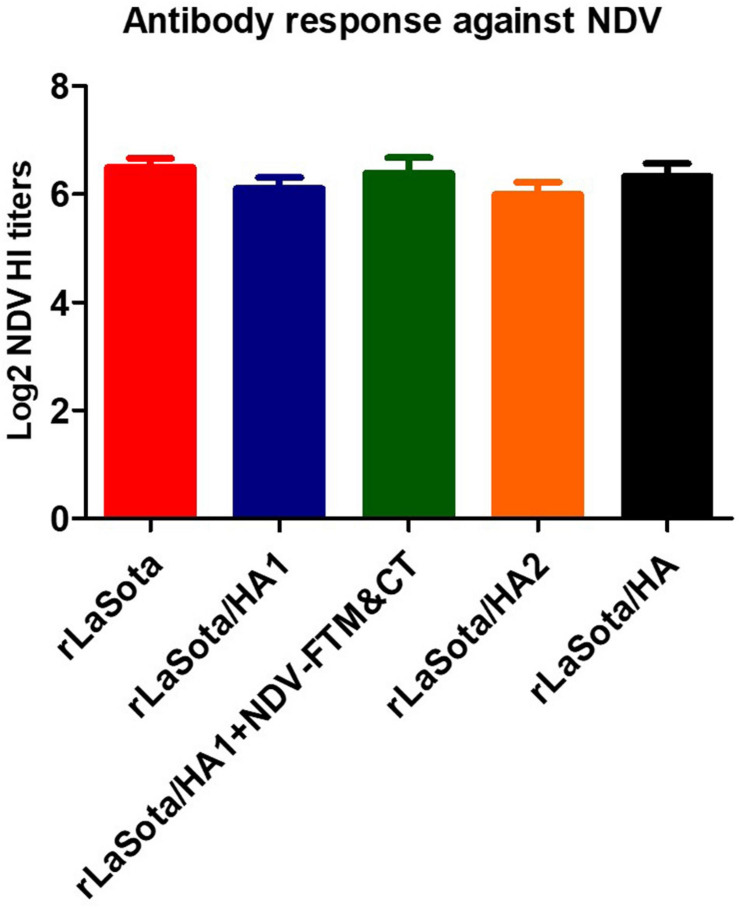
Antibody response against NDV. SPF chicks were immunized at one day old and sera were collected at day 21 after immunization. HI antibody titers induced against NDV strain LaSota in collected sera were assessed by HI assay using the protocol of OIE. Serum titers are expressed as reciprocal Log2 dilution.

The results showed that comparable HI titers against NDV strain LaSota were detected in sera collected from chickens of five groups (*P* > 0.05) (Figure 6). The data were statistically analyzed by the One-Way-ANOVA analysis (Tukey test) among five groups.

## Discussion

This study aimed to determine the individual contributions of HA1 and HA2 subunits of H5N1 HPAIV HA protein in the induction of neutralizing antibodies and protection in chickens. rNDV was used as the expression vector, because NDV infects the respiratory tract just as HPAIV does and has been previously used to express HPAIV HA protein (Veits et al., 2006; Nayak et al., 2009; Kim and Samal, 2016). Chickens are highly susceptible to HPAIV infection; therefore, are an ideal system for this study. Our results showed that the intact HA protein induced a neutralizing antibody response and provided complete protection against death following a lethal homologous challenge. Whereas, HA1 or HA2 subunit neither induced significant levels of a neutralizing antibody response nor provided protection following a lethal homologous challenge. These results suggest that the epitopes responsible for the induction of protective immunity are present on both the HA subunits in their native conformation, but their native conformation is lost when the two subunits are separated.

The intact HA protein and HA1 fused with NDV F TM and CT domains were detected at high levels in DF1 cells by both Western blot and IFA. The HA2 protein was also detected in DF1 cells by IFA at a high level and by Western blot at a lower level, but the HA1 protein was not detected in DF1 cells by IFA and detected by Western blot at a very low level. We also evaluated expression of these proteins in the egg and incorporation of them into NDV particles. Among the four proteins, the intact HA protein was detected in the egg at a high level and incorporated into the NDV envelope efficiently; the HA2 protein was detected in eggs at a low level and incorporated into the NDV envelope inefficiently. HA1 with NDV F TM and CT was detected at very low level in the egg and showed very little incorporation, and HA1 was not detected in the egg and did not incorporate into the NDV virion. Furthermore, the size of detected HA1 and HA2 proteins either in cell or in egg are different from the size of detected bands for the cleaved HA1 and HA2 from whole HA protein, respectively. Therefore, among HPAIV antigens, only whole HA was detected at a high level by anti HPAIV serum regardless the environment of expression and incorporated efficiently into NDV envelop. These results suggest that separation of HA1 and HA2 subunits led to a change in the conformational structure of immunoreactive epitopes on HA1 and HA2 subunits. The addition of NDV F TM and CT domains might have restored the conformation of some of the immunoreactive epitopes when protein was expressed in DF1 cells, but not in the egg, which may be because some cellular factors needed for folding are absent in the egg. Oligomerization studies also showed that HA1, HA2, and HA proteins formed oligomers, suggesting that the inability of HA1 to react with H5N1 antiserum was not due to absence of oligomerization (Supplementary Figure 3).

The result of the protective efficacies of rNDVs expressing HA1, HA2, or HA protein in one-day-old chicks showed that rNDV expressing the intact HA protein provided complete protection against HPAIV infection in chickens, while rNDV expressing HA1 or HA2 subunit were not protective. Our finding that the HA provided complete protection from lethal challenge was not unexpected, because it is well established that the HA protein contains the major protective antigens of HPAIV (Veits et al., 2006; Nayak et al., 2009). However, our results showed that when separated the HA1 or HA2 subunit alone does not provide protection in chickens. These results suggest that the native conformation of HA is maintained when the HA1 and HA2 subunits are held together by disulfide bonds. The important protective epitopes are only present on HA in its native conformation. But when HA1 and HA2 subunits are separated, the conformations of these two proteins are changed, leading to the loss of the protective epitopes. It is also possible that some protective epitopes on HA protein are formed by residues from both HA1 and HA2 subunits and hence these epitopes are lost when the two subunits are separated.

The neutralizing antibodies against a H5N2 LPAIV were detected only in serum samples of chickens immunized with rNDV expressing the whole HA protein. Our results showed that the HA1 and HA2 subunits, when separated, failed to induce neutralizing antibodies. These results suggest that the conformational changes caused by the separation of HA1 and HA2 subunits might be the reason for the loss of neutralizing epitopes located on HA1 and HA2 subunits. Our results provide indirect evidence that the disulfide bond between HA1 and HA2 or some other interactions between key residues of HA1 and HA2 is necessary to maintain the native conformation of HA protein (Palese and Shaw, 2013; Wang et al., 2015). This is consistent with outcomes of an earlier study which showed that the interaction between HA1 and HA2 subunits affects HA protein stability and AIV infectivity (Wang et al., 2015). Interestingly, rNDV expressing HA1 subunit fused with NDV F TM and CT also did not induce neutralizing antibodies or provide protection, but somehow resulted in high levels of expression of HA1 detected by Western blot analysis and IFA only in DF1 cells. This result suggests that the addition of NDV F TM and CT leads to conformational change of HA1 and exposure of non-neutralizing epitopes, which were recognized by AIV antibodies in Western blot and IFA. However, the presence of NDV F TM and CT was not able to compensate for the lack of HA2 subunit required for correct folding, incorporation into the NDV envelope, and induction of protective immunity. Previous studies have shown that HA1 and HA2 subunits of H5N1 HPAIV (Gao et al., 2006) and stem fragment of H1N1 (Steel et al., 2010; Mallajosyula et al., 2014; Yassine et al., 2015) provided protection and induced antibodies in mouse and/or ferret models. Although mice and ferrets are excellent animal models to study human influenza virus pathogenesis and transmission, they may not be sufficiently permissive to evaluate the protective efficacy of human influenza virus vaccines (Thangavel and Bouvier, 2014; Zeng et al., 2019).

The antibodies against NDV were detected at similar levels in immunized chickens of all groups, indicating that all the recombinant viruses grew at the same level in chickens. The level of antibodies induced against the NDV backbone was higher than antibodies detected against AIV for two reasons. Firstly, for NDV, a homologous virus was used in HI assay for the detection of antibodies against NDV, while for HPAIV, a heterologous LPAIV (H5N2), with about 90% amino acid identity with the HA protein of recombinant vaccine, was utilized to detect HI and neutralizing antibody titers against H5N1 HPAIV. Previous studies have shown that the antibody titers assayed against a heterologous H5 HPAIV were lower compared to the homologous H5 HPAIV virus (Swayne et al., 2015; Jang et al., 2018). Secondly, for HPAIV, antibodies induced against HA protein contribute to HI and VN assays. But for NDV, the antibodies induced against heamagglutininin–neuraminidase (HN) protein are detected by HI test and antibodies induced against F and HN proteins contribute to the VN assay (Samal, 2019).

## Conclusion

Therefore, a H5N1 vaccine construct incorporating either HA1 or HA2 subunit may not provide protection against HPAIV challenge in chickens. Our results showed that immunization with HA1 or HA2 alone neither induced serum neutralizing antibodies nor prevented death following challenge. Immunization with HA protein is necessary for complete protection against HPAIV.

## Data Availability Statement

All datasets generated for this study are included in the article/Supplementary Material.

## Ethics Statement

The animal study was reviewed and approved by Institutional Animal Care and Use Committee (IACUC), University of Maryland.

## Author Contributions

SS and ES conceived and designed the experiments, analyzed the data, and wrote the manuscript. ES built the constructs and *in vitro* characterization experiments and performed the immunization and animal experiments in Biosafety level 2 facilities. AP and BV performed the HPAIV challenge and animal experiments in Biosafety level 3 plus facilities. SS contributed reagents, materials, and analysis tools. All the authors reviewed the manuscript.

## Conflict of Interest

The authors declare that the research was conducted in the absence of any commercial or financial relationships that could be construed as a potential conflict of interest. The reviewer, DR, declared a past co-authorship with one of the authors, SS, to the handling editor.
