# Supplementary material for: Contributions of HA1 and HA2 Subunits of Highly Pathogenic Avian Influenza Virus in Induction of Neutralizing Antibodies and Protection in Chickens
Source: Front Microbiol. 2020 Jun 5;11:1085. doi: 10.3389/fmicb.2020.01085 (PMC7291869; doi:10.3389/fmicb.2020.01085)
Supplement: Supplementary file 1 [file Data_Sheet_1.pdf]

Supplementary Material

Supplementary Figures

2A (upper panel)

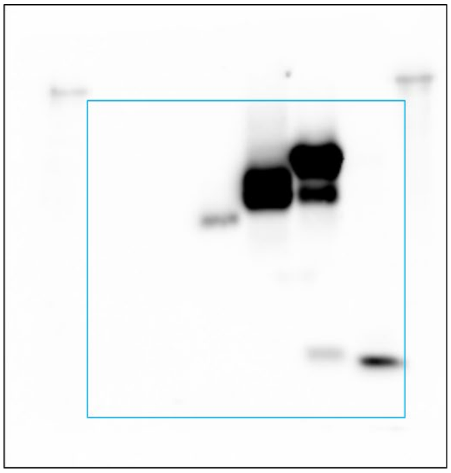

2B (upper Panel)

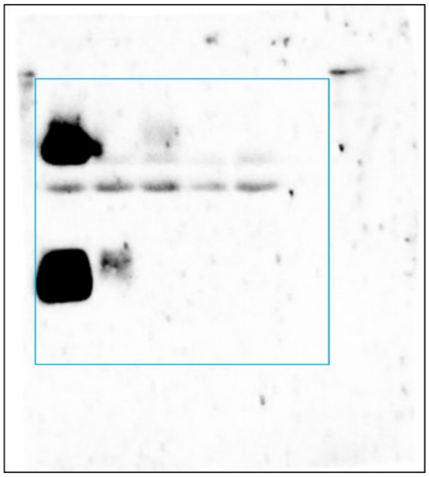

2A (lower panel)

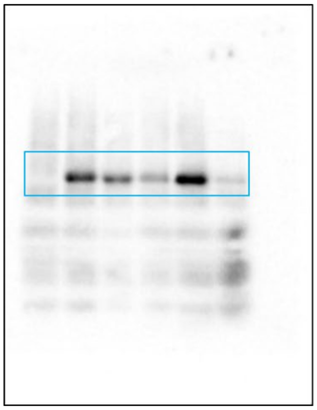

2B (lower panel)

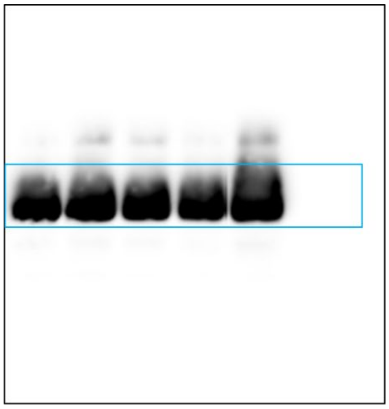

Supplementary Figure 1. Full length of western blot gels which the cropped versions of them were shown in the main text. Black boxes show the full length of the gels for Fig. 2A and B- upper and lower panels. The cropped areas are shown in blue boxes.

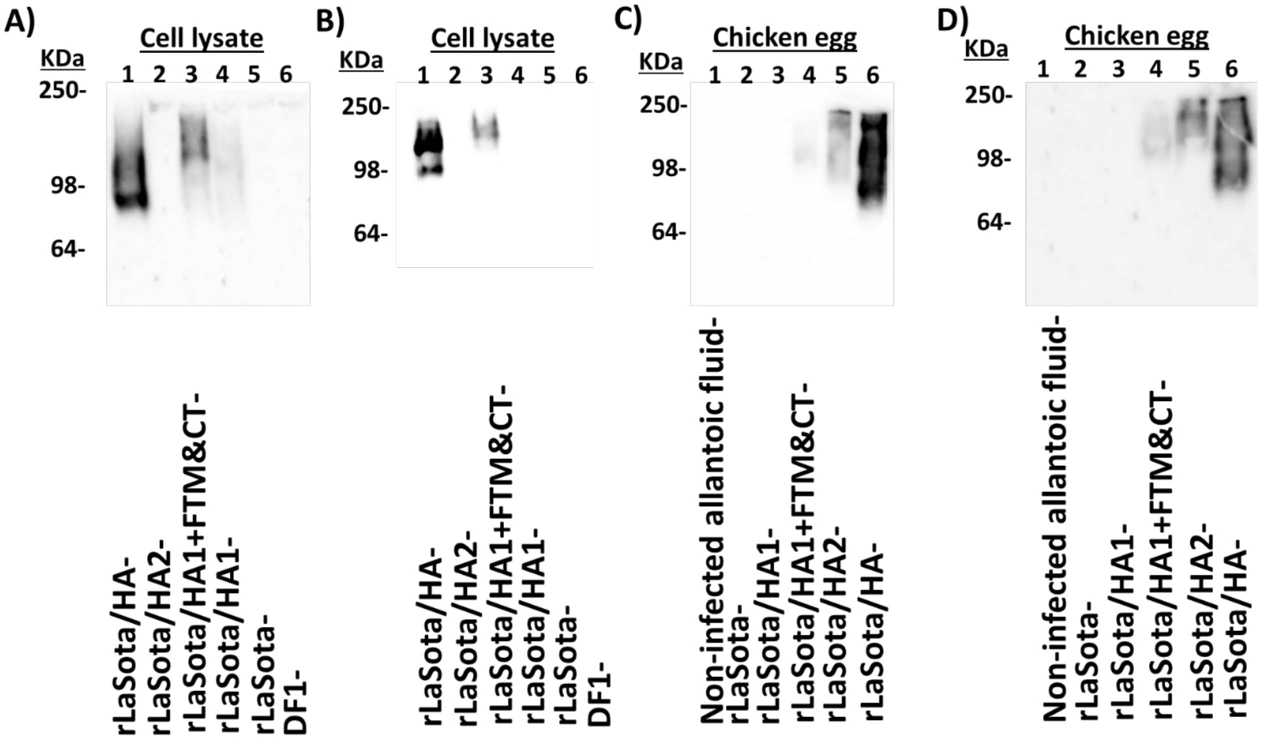

Supplementary Figure 2. The conformational structure of HA1, HA2 and HA proteins. The expression of HA1, HA2 and HA proteins in DF1 cell lysates by rNDV were detected using non-reducing and non-denaturing conditions. The monolayer of DF1 cells were infected with rLaSota, rLaSota/HA1, rLaSota/HA1+NDV F TM & CT and rLaSota/HA. The infected cells were collected 24 hours after infection with 100 µl of 2X non-reducing laemmli sample buffer (BioRad) per each well of 12-well tissue culture plate. Six µl of each sample was loaded into a 7.5% native electrophoreses gel (without SDS) separating gel (A) or was loaded into a 7.5% native electrophoreses gel (without SDS) separating gel plus 4% native electrophoreses gel (without SDS) stacking gel (B). The equal volume of 2X non-reducing laemmli sample buffer (BioRad) also was added to each sample, prepared using the protocol described for detection of expression of proteins in chicken eggs and incorporation of proteins into rNDV particles. Six µl of each sample was loaded into 7.5% native electrophoreses gel (without SDS) separating gel (C) or was loaded into 7.5% native electrophoreses gel (without SDS) separating gel plus 4% native electrophoreses gel (without SDS) stacking gel (D). The running buffer without SDS was used to run the samples through the electrophoresis gels. The same polyclonal chicken anti H5N1 HPAIV serum which was used for the detection of expression and incorporation of HPAIV proteins was utilized to determine the structural conformations of HA1, HA2 and HA proteins.

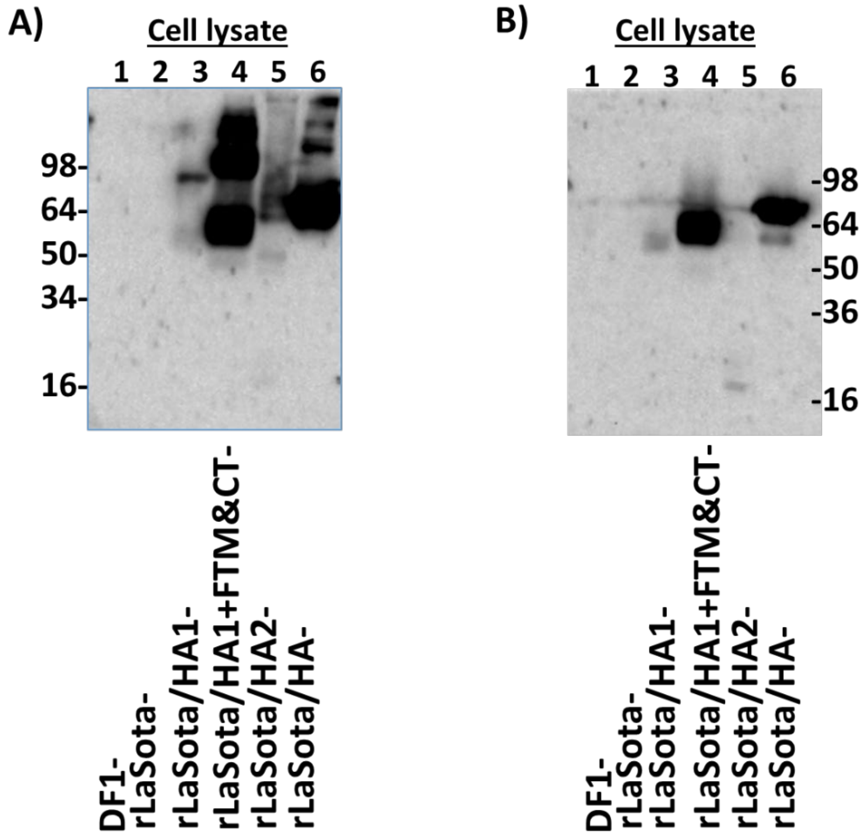

Supplementary Figure 3. The oligomerization of HA1, HA2 and HA proteins. The monolayer of DF1 cells were infected with rLaSota, rLaSota/HA1, rLaSota/HA1+NDV F TM & CT and rLaSota/HA. The infected cells were collected 24 hours after infection with 100 µl of 6X non-reducing laemmli sample buffer per each well of 12-well tissue culture plate. Seven µl of each sample was loaded into a 10% SDS electrophorese after heating (non-reducing and denaturing conditions) (A). 2-mrcaptoethanol was added to of each sample and seven µl of each sample also was loaded into another 10% SDS electrophorese after heating (non-reducing and denaturing conditions) (B). A polyclonal chicken anti H5N1 HPAIV serum was used to determine the expression and oligomerization of HA1, HA2 and HA proteins. Lines 1 to 6 in panels A and B represents DF1 cells, rLaSota, rLaSota/HA1, rLaSota/HA1+NDVF TM&CT, rLaSota/HA and rLaSota/HA2, respectively.
